# I Am Not a Scientist, I Am a Number

**DOI:** 10.1371/journal.pcbi.1000247

**Published:** 2008-12-26

**Authors:** Philip E. Bourne, J. Lynn Fink

**Affiliations:** Skaggs School of Pharmacy and Pharmaceutical Sciences, University of California San Diego, La Jolla, California, United States of America; Constellation Pharmaceuticals, United States of America

We suspect many of our readers will be familiar with the cult TV show *The Prisoner*, in which actor Patrick McGoohan had his identity taken away by unknown assailants for unknown reasons, and his pleas of “I am not a number, I am a person” (http://www.youtube.com/watch?v=29JewlGsYxs&feature=related) were greeted with variants of “whatever you say, number six.” We would suggest that, as scientists, we are in a situation where the opposite will soon be true, at least for the purposes of scientific scholarship. Scientists will want to be assigned a number, or at least a unique identifier. Why?

Imagine a time when you and your complete scholarly output—papers, grant applications, blog posts, etc.—could be identified online and in perpetuity and returned in a variety of easy-to-digest ways. While ego comes into it as a driver to make this happen, measuring scientific career advancement is something that lacks good metrics in a digital world. Unless one has a truly unique name, applying such a metric is not possible now. Even with a unique name, what is the guarantee that all of our scholarly output will be captured by one source of that information? In the end, we as individuals are the only ones who reliably track our scholarly output. This situation is beginning to change, and, as we shall see, new metrics have the promise of much more than simply returning references to our collective life's work as currently described by research papers, research proceedings, books, and book chapters. Although even a complete and current resume generated on demand would be a big step, if it could be returned in a variety of formats for a variety of purposes. These complete resumes are something many of us spend endless hours generating.

The idea of having our scholarly output properly characterized is not out of reach, since the articles we write are already identified uniquely by a Digital Object Identifier (DOI; discussed further below). A book or journal is identified by an ISBN, and citations are identified by PubMed identifiers, and so on. The ideas discussed here simply take this identification process for individual publications and citations to the point of providing unique descriptors for each author and to uniquely identify *all* of each author's scholarly work.

## Unique Identifiers

Initiatives such as OpenID (http://openid.net/) and ResearcherID (http://www.researcherid.com/), if they catch on in the scientific community, promise to provide us with unique identifiers. The beginnings of what might lead to the adoption of a professional identifier did not start in the scientific, i.e., professional community, but in online social communities. Those of us weary of the need to remember multiple usernames and passwords to all of the Web sites we access on a regular basis can see the merits of an OpenID, provided the integrity of our information can be maintained. Much has already been written about OpenID, including adoption by Google and Microsoft, among others, and we will just briefly introduce it here. More relevant here is how and if publishers and scientists at large will embrace the concept and what it means to us as scholarly communicators.

The basics of an OpenID system are as follows. A user requests and is granted an OpenID from an ID provider. In so doing, they create a profile for themselves, which is in a standard format and can be exported and shared by many other sites, provided the owner authorizes that sharing. Thus, the intent is that an OpenID would eventually work on all the Web sites that the user typically visits, assuming those sites adopt the standard. Attempting to log onto such a site triggers an authentication process with the ID provider and access to the site if the authentication requirements are met. Consider how this might work in electronic publishing. Authors would already have or be assigned OpenIDs—a key issue that we will come to in a moment—and the papers they author would have that OpenID assigned to them. Thus, a unique 1-to-1 correspondence is established that is not possible when using a human name, assuming the OpenID namespace is kept unique. If each publisher were to assign their own separate, unique ID to each author, the value to the author would be minimal. However, we are confident that publishers will come together in some way behind a specific system, if not OpenID then something they collectively agree upon. We say this based on the fact that publishers have done this already for individual pieces of published work. Most Science, Technical, and Medical (STM) publishers have embraced the use of DOIs, which provide a resolver mechanism to find the definitive reference to a piece of scholarly work. The DOI provides the original reference to the scholarly work in a virtual world where many copies and derivatives may have been created. The idea of creating an exact mapping between the author and a piece of their published work as it exists in cyberspace, and being able to resolve that mapping, is a simple extension of the same idea. If you as an author can be uniquely identified, you can in principle be more accurately mapped to all of your scholarly output if each item of that output is tagged with your identifier. There are several ifs associated with this concept—*if* the idea of an OpenID will take off, *if* publishers embrace it, *if* scientists agree to be identified in this way. Success will most likely come if momentum builds and if applications that use the concept can be shown to benefit the consumer. This would seem to be the approach that Thomson Reuters is taking.

Thomson Reuters has introduced ResearcherID, which begins to make apparent the promise of unique identifiers, in this case because it is linked directly to the author's scholarly output using the ISI Web of Knowledge, also a Thomson Reuters product. The use of this system is currently by invitation only, and it is not clear how access and cost will be defined in the future. By visiting their Web site (http://www.researcherid.com), you will see that computational biologists (including ourselves) are already some of the most active participants. Since for many of us the Web of Knowledge only covers a part of our scholarly output, this, too, is limiting, but it begins to illustrate the possibilities. It should be said that none of these current identification possibilities are a function of authors having a unique identifier across the scientific industry and community; it simply makes returns more accurate, if not complete. An alternative or additional possibility is that we could each be assigned our own DOI which we could use to relate to OpenID, ResearcherID, and any new ID schemas.

Having accepted the notion that you will be represented in cyberspace by a single unique identifier, the first step is to define your profile associated with that identifier. Many of us have done this in many different contexts many different times—LinkedIn, Facebook, a journal's Web site where we have submitted a paper, etc. The idea of having all the relevant information pulled from a central registration database rather than entering it each time is compelling, but also raises security concerns. For us, the idea of keeping one centralized copy of our resume (part private, part public) is desirable even if the systems do not quite support that idea yet. Similarly, we may want to share different parts of our profile, for example, personal interests, depending on the nature of the Web site (social or professional networking). Another advantage is the removal of dependence on a professional name. Many people change or alter their name for various reasons, and if this is done after a publication record has already begun, it can be difficult for others to follow a single author's work.

If you sign up for ResearcherID, or just browse someone's profile, the promise of an OpenID begins to emerge. Immediately, all that Web of Knowledge has to offer is available papers, number of times cited, etc. You can review citation metrics, for example, the number of times your papers have been cited per year, who has cited them the most, where they are from, and so on. You can create an icon for yourself that can be embedded in any Web page, so, for example, your latest papers could be presented to anyone who happened to mouse over your name on a Web page. Your scholarly output is laid bare—at least that part of it that ISI Web of Knowledge keeps track of. ResearcherID illustrates the promise, but it is proprietary. It will be interesting to see if an open solution surfaces. Certainly an OpenID could be associated with indexed content in Google Scholar, creating an open equivalent that covers a broader set of literature than the Web of Knowledge.

Putting aside the issue of open versus closed, with an ID you are now uniquely identified in cyberspace, and so in principle anything that is associated with your unique identifier can be returned. We will see the promise of that in a minute, but let us first contrast these possibilities to the metrics we have currently.

## Metrics

There is much debate about the value of impact factors assigned to journals and the impact of individual research articles, and, of course, the H factor assigned to authors. The weaknesses of these systems have been widely discussed, yet their use persists as they are currently the only widely and easily obtainable metrics, but this can change. One of us (PEB) likes to cite his own situation to highlight issues with current metrics and why we think the situation should change. PEB has a paper that has been cited more than 6,000 times, but he suspects hardly anyone has ever read it; it is a reference to a commonly used database he helped develop. How should one rate that versus another of his papers that he believes has contributed to a new area of biological study yet has only been cited 50 times? Similarly, he writes Editorials and Perspectives that stimulate lots of download and discussion, but get cited rarely; what is their relative impact on science? Some of us write blogs and other postings that are widely read, so how do we measure the impact of that kind of discourse? How does one measure the impact of adding an entry to a public database, a page to a wiki, or reviews of manuscripts? There are no simple answers to these questions. What is clear is that even beginning to develop some kind of new personal impact factor requires that all these kinds of materials be identified in cyberspace and accrued into some composite value. Assigning each of us a unique identifier, and tagging all that we produce with that identifier, is requisite for accurately finding that information in cyberspace.

Here is a simple suggestion and a call to action to get the ball rolling. Open access publishers require authors to use or obtain an OpenID so that they are identified uniquely with their papers. They then make available to the authors download statistics for their papers on a regular basis (some publishers do this already). Over time, we anticipate that authors will start quoting these numbers in the same way they do the number of times the paper is cited. Some will say this is not the same since downloading a paper is not the same as citing it. Partially true, but can we say we have fully read all the papers we cite? At least we are introducing a new metric into the mix. These download statistics can also be associated with the papers themselves and made public. Neither downloads nor citations necessarily mean a high quality paper, as poor, wrong, and controversial papers are subject to citation and download, too.

Taking the idea of new metrics a step further, here is a straw man metric for you to comment on and improve. We realize at the outset that this metric will likely aggravate a number of readers. Why should we choose to quantify our scholarly output in this way? Our answer would be: so as to be fair and to create a reward that is a reflection of what is important to impart, which is more than just the contents of a scientific paper. We refer to this metric as the Scholar Factor (SF).

We define for each scientist a Scholar Factor (SF) as follows:
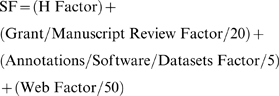
where:

H Factor is as it is now—the number of papers cited more than H times—thus, an H factor of 20 indicates that an author has 20 papers cited more than 20 times. An H factor derived from Google Scholar data (assuming an author could be uniquely identified) will likely be higher than that currently derived from other citation sources, since for many scientists more scholarly output is indexed by Google Scholar than by any other single citation index.

Grant/Manuscript Review Factor is the accumulative number of authenticated (we will get to authentication in a minute) grant and paper reviews you have done (data provided to the grant funding agencies and journals); 20 reviews increases your SF by 1.

Annotations/Software/Datasets Factor is the accumulative number of authenticated entries you have made in a public database, for example, microarray datasets, gene sequences, macromolecular structures, or software entries you have added to an open access archive. If n scientists were involved in making the entry, you get 1/n of an entry; 5 entries increase your SF by 1. Annotation of a genome should likely count more than a gene, and so the amount of work performed also needs to be included here, but you get the idea.

Web Factor is the number of authenticated blog posts, wiki postings, etc., you make that show x or more links to them (a measure of their value), where x is to be determined; 50 entries increases your SF by 1.

Sites that accept scholarly communications will be asked to be authenticators and to provide a standard mechanism to automatically authenticate entries. An authenticated submission to a participating resource will provide a token back to the submitter, which forms the basis for increasing their SF. Why would a database or wiki go to the trouble of developing software to do and track this process? Perhaps through demand from anxious scientists who have long wanted to make blog posts or annotate database records but felt there was no reward in doing so. Perhaps, because if they do not, scientists will make their scholarly contributions elsewhere? Ideally, there should be a central resolver for authenticated tokens in the same way as there is a resolver for DOIs to get to the definitive source of the literature reference, except in this case it would be to get to the original source author of the material posted.

We have to admit that this would require significant change to the scholarly process, which is not going to happen overnight in a conservative field, but we are interested in your comments on the concept of a Scholar Factor, whatever the form it takes.

As much as we like watching reruns of *The Prisoner*, we have to say it is time we were assigned a number as we attempt to quantify scientific progress both in general and for the individual in a virtual world in which scientific progress is based on more than the impact of a journal article. What do you think? Who knows—perhaps some day you will be rewarded for your time, energy, and intellect that go into a thoughtful response to this Perspective.

